# Checkpoint Kinase 1 Activation Enhances Intestinal Epithelial Barrier Function via Regulation of Claudin-5 Expression

**DOI:** 10.1371/journal.pone.0145631

**Published:** 2016-01-04

**Authors:** Akihiro Watari, Maki Hasegawa, Kiyohito Yagi, Masuo Kondoh

**Affiliations:** Laboratories of Bio-Functional Molecular Chemistry, Graduate School of Pharmaceutical Sciences, Osaka University, Suita, Osaka, Japan; University Hospital Hamburg-Eppendorf, GERMANY

## Abstract

Several stressors are known to influence epithelial tight junction (TJ) integrity, but the association between DNA damage and TJ integrity remains unclear. Here we examined the effects of daunorubicin and rebeccamycin, two anti-tumor chemicals that induce DNA damage, on TJ integrity in human intestinal epithelial cells. Daunorubicin and rebeccamycin dose-dependently enhanced transepithelial electrical resistance (TER) and decreased flux of the 4 kDa FITC-dextran in Caco-2 cell monolayer. Daunorubicin- or rebeccamycin-induced enhancement of the TJ barrier function partly rescued attenuation of the barrier function by the inflammatory cytokines TNF-α and IFN-γ. Daunorubicin and rebeccamycin increased claudin-5 expression and the product was distributed in the actin cytoskeleton fraction, which was enriched with TJ proteins. Caffeine, which is an inhibitor of ataxia telangiectasia mutated protein (ATM) and ataxia telangiectasia mutated and Rad3-related protein (ATR), and the Chk1 inhibitor inhibited the TER increases induced by daunorubicin and rebeccamycin, whereas a Chk2 inhibitor did not. Treatment with Chk1 siRNA also significantly inhibited the TER increases. Induction of claudin-5 expression was inhibited by Chk1 inhibitor and by siRNA treatment. Our results suggest that Chk1 activation by daunorubicin and rebeccamycin induced claudin-5 expression and enhanced TJ barrier function in Caco-2 cell monolayer, which suggests a link between DNA damage and TJ integrity in the human intestine.

## Introduction

Cells respond to damage to their DNA or interference with their DNA replication by activating genome surveillance pathways such as cell cycle checkpoints. Although the response to DNA damage comprises a complex network of signals, overall the network can be considered to contain three sequential steps: sensing of the damage, transduction of the damage signal, and execution of cell cycle arrest, apoptosis, and DNA damage repair [1, 2]. DNA damage is recognized by sensor proteins such as ataxia telangiectasia mutated protein (ATM) and ataxia telangiectasia mutated and Rad3-related protein (ATR). When ATM and ATR are recruited to sites of DNA damage, they activate checkpoint kinase 1 (Chk1) and checkpoint kinase 2 (Chk2), respectively, which regulate the function of downstream effector proteins such as p21, Cdc25A, and cyclin-dependent kinases [1]. Depending on the extent of the DNA damage, cells then either go into cell cycle arrest to allow time for repair or go into apoptosis [3]. This orderly, accurate response to DNA damage is essential to maintain the genetic stability of cells.

Epithelial tissue is a physical barrier that separates the internal and external environments. Adjacent epithelial cells are joined by junctional complexes such as tight junctions (TJs), adherens junctions, desmosomes, and gap junctions. TJs are located at the apical end of the basolateral membrane between polarized epithelial cells [4]. TJs contribute to maintaining a distinct internal environment by functioning as the primary barrier to the intrusion of external agents and controlling the diffusion of solutes through the intercellular space [5, 6]. TJs are composed of transmembrane proteins (e.g., claudin, occludin, tricellulin, junction adhesion proteins [JAMs]) and cytoplasmic scaffolding proteins (e.g., zonula occludens [ZO]-1, ZO-2, ZO-3; cingulin). Claudins are the major structural and functional components of TJs. Claudins are tetra-transmembrane proteins with a molecular weight of around 23 kDa, and the claudin family comprises 27 members in mammals [7, 8]. Claudins can be classified functionally into those with sealing functions (e.g., claudin-1, -3, -5, -11, -14) and those with channel-forming functions (e.g., claudin-2, -10, -15). The functions of other claudins, such as claudin-4, -7, -8, and -16, remain unclear as their effects on epithelial barriers are inconsistent. The expression profiles and barrier functions of these claudins provide tissues their barrier-specific properties [9], and claudin dysfunction is associated with the development of various diseases [10]. A number of agents have been reported to modulate TJ barrier function [11–13], making TJ modulation a potential therapeutic strategy for the treatment of diseases in which TJ integrity is compromised [10, 11, 14]. However, large-scale compound screening for TJ or claudin modulators is rarely undertaken.

Previously, we developed a cell-based reporter system for the detection of claudin-4 expression by using a functional claudin-4 promoter, and identified several claudin-4 modulators from among 86 chemicals used as food additives [15] and 2642 other validated compounds (Library of Pharmacologically Active Compounds^1280^ and Prestwick Chemical Library) [16]. We found that the anti-tumor agents daunorubicin and rebeccamycin regulated claudin expression in mammary gland epithelial cells [16]. Daunorubicin exerts its anti-tumor activity mainly by inducing DNA damage in cancer cells through intercalation between DNA base pairs or by interfering with DNA topoisomerases [17]. Rebeccamycin also induces DNA damage by inhibiting topoisomerases [18]. Although much effort has been made to understand the anti-tumor activities of daunorubicin and rebeccamycin, the correlation between daunorubicin- and rebeccamycin-mediated DNA damage and TJ integrity remains unknown. Here we evaluated the effects of daunorubicin and rebeccamycin on TJ integrity, and investigated the association between DNA damage signaling and the components of TJs in human intestinal cells.

## Materials and Methods

### Chemicals

Daunorubicin, cisplatin, and mitomycin C were purchased from Wako Pure Chemical Industries (Osaka, Japan). Rebeccamycin and caffeine were purchased from Sigma-Aldrich (St. Louis, MO, USA). SB218078 and Chk2 inhibitor were purchased from Calbiochem (San Diego, CA, USA).

### Antibodies

Rabbit anti-claudin-1 polyclonal antibody (pAb), mouse anti-claudin-2 monoclonal antibody (mAb), rabbit anti-claudin-3 pAb, mouse anti-claudin-4 mAb, mouse anti-claudin-7 mAb, mouse anti-occludin mAb, rabbit anti-tricellulin pAb, rabbit anti-ZO-1 pAb, rabbit anti-ZO-2 pAb, and rabbit anti-JAM-A pAb were purchased from Invitrogen (Carlsbad, CA, USA). Rabbit anti-claudin-5 pAb and mouse anti-β-actin mAb were purchased from Abcam (Cambridge, MA, USA) and Sigma-Aldrich (St. Louis, MO, USA), respectively. Rabbit anti-ATR pAb, anti-ATR pS428 pAb, mouse anti-Chk1 mAb, and rabbit anti-Chk1 pS345 pAb were purchased from Cell Signaling Technology (Beverly, MA, USA). Goat anti-Rabbit IgG peroxidase-conjugated antibody and goat anti-mouse IgG peroxidase-conjugated antibody were purchased from Millipore (Bedford, MA, USA). Alexa Fluor 488 goat anti-rabbit IgG was purchased from Molecular Probes (Eugene, OR, USA).

### Cell culture

Human colorectal adenocarcinoma cell lines Caco-2 (HTB-37) and T84 were obtained from American Type Culture Collection. Caco-2 cells were cultured in Eagle's minimum essential medium (Nissui, Japan) supplemented with 10% FBS under 5% CO_2_ at 37°C. The passage number used for the experiments was between 20 and 35. T84 cells were cultured in Dulbecco's modified Eagle's medium—Ham’s F-12 (Nissui, Japan) supplemented with 10% fetal bovine serum.

### Measurement of epithelial barrier function

Epithelial barrier function was assessed by measuring transepithelial electrical resistance (TER) and flux of fluorescein isothiocyanate (FITC)-labeled dextran in human intestinal cell monolayers. In the TER assay, Caco-2 or T84 cells were seeded in Transwell chambers (diameter, 6.5 mm; pore size, 0.4 μm; Corning, MA, USA) at a density of 6 × 10^4^ or 10 × 10^4^ cells/well, respectively, and cultured for 10 to 14 days. After TER values had plateaued, daunorubicin or rebeccamycin was added to the upper and lower chambers, and TER value was measured with a Millicell-ERS epithelial volt-ohmmeter (Millipore Corporation, Billerica, MA, USA).

In the paracellular tracer flux assay, Caco-2 cells were cultured in Transwell chambers (diameter, 6.5 mm; pore size, 0.4 μm; Corning, MA, USA) for 3 days at a density of 6 × 10^4^ cells/well. After equilibration with P buffer (10 mM HEPES [pH 7.4], 1 mM sodium pyruvate, 10 mM glucose, 3 mM CaCl_2_, and 145 mM NaCl), 100 μM FITC-labeled dextran with a molecular mass of 4 kDa (4-kDa FITC—dextran) in P buffer was added to the upper chamber. After incubation for 1 h, the concentration of 4-kDa FITC—dextran in the lower chamber was determined by measuring fluorescence with a TriStar LB 941 microplate reader (Berthold Technologies, Wildbad, Germany).

The effect of TNF-α and IFN-γ on barrier function in Caco-2 monolayer was also determined by using a TER assay. Caco-2 monolayers were cultured for 10 to 14 days in Transwell chambers and then treated with recombinant human TNF-α (10 ng/mL) and IFN-γ (10 ng/mL) (R&D Systems, Minneapolis, USA), which were added to the lower chamber. Monolayers incubated with medium containing vehicle were used as the control.

### Cytotoxicity assay

Caco-2 cells were seeded in Transwell chambers (diameter, 6.5 mm; pore size, 0.4 μm; Corning) at a density of 6 × 10^4^ cells/well and cultured for 10 to 14 days. After being treated with daunorubicin or rebeccamycin on the apical and basolateral sides for 24 h and 48h, cytotoxicity was assessed by measuring lactate dehydrogenase (LDH) release from Caco-2 cells by using an LDH cytotoxicity test kit (Wako, Osaka, Japan) in accordance with the manufacturer’s instructions. Treatment with 0.2% Tween-20 diluted in PBS was used as a 100% lysis control. Optical density was measured at 570 nm with a TriStar LB 941 microplate reader (Berthold Technologies).

### Quantitative reverse transcriptase-PCR (qRT-PCR) analysis

Claudin-1, -2, -3, -4, -5, -7, -8, 10, -11, -12, -14, -15, occludin, tricellulin, JAM-A, and ZO-1 mRNA levels in cells treated with vehicle, daunorubicin, or rebeccamycin for 24 h were analyzed by means of qRT-PCR analysis. After treatment with vehicle, daunorubicin, or rebeccamycin, Caco-2 cells were washed with PBS and total RNA was extracted with TRIzol reagent (Invitrogen). Total RNA (3 μg) was reverse transcribed to cDNA with a cDNA synthesis kit (Roche, Mannheim, Germany) in accordance with the manufacturer’s instructions. The resulting cDNA was used for qRT-PCR analysis. Sequences of the primers used are listed in S1 Table. qRT-PCR was performed with SYBR Premix Ex Taq II (Takara, Shiga, Japan) and an Applied Biosystems StepOne Plus system (Applied Biosystems, Foster City, CA). Relative quantification was performed against a standard curve, and the values were normalized to those for the housekeeping gene glyceraldehyde 3-phosphate dehydrogenase.

### Preparation of cell lysate and immunoblot analysis

Caco-2 cells were seeded in Transwell chambers (diameter, 24 mm; pore size, 0.4 μm; Corning) at a density of 7.5 × 10^5^ cells/well and cultured for 10 to 14 days. Cells were lysed with 1% Triton-X buffer (50 mM Tris—HCl [pH 7.4], 1.0% Triton X, 5 mM EGTA) containing protease inhibitor cocktail (Sigma-Aldrich). Cell lysates were centrifuged at 15,600*g* for 4 min at 4°C to sediment the high-density, actin-rich fraction. The pellet was suspended in lysis buffer (10 mM Tris—HCl [pH 7.4], 0.3% SDS) containing protease inhibitor cocktail. Whole cell lysate was prepared in lysis buffer. Cell lysates were run on an SDS—polyacrylamide gel and electroblotted onto a PVDF membrane. The membranes were incubated successively with antibodies against claudin-1, -2, -3, -4, -5, -7, occludin, tricellulin, ZO-1, ZO-2, JAM-A, and β-actin, and then with a horseradish peroxidase-conjugated anti-rabbit or -mouse IgG antibody. Reactive bands were detected with an enhanced chemiluminescence reagent (GE Healthcare, Buckinghamshire, UK), and signals were visualized with an ImageQuant LAS4010 imaging system (GE Healthcare).

### Construction of retroviral vector and virus production

pCX4, a Moloney murine leukemia virus-based retroviral vector containing the puromycin resistance gene as a selectable marker, was constructed by Akagi et al. [19]. pCX4-claudin-5 was constructed by inserting full-length human claudin-5 cDNA into the multi-cloning site of the pCX4 vector. Phoenix-A cells were transfected with pCX4 or pCX4-clauidn-5 constructs by using X-tremeGENE HP (Roche Diagnostics, Mannheim, Germany) in accordance with the manufacturer’s protocols. Two days after transfection, culture supernatants were collected and stored at −80°C until use.

### Transfection of siRNA

Caco-2 cells were cultured in 6-well plates (Corning). To suppress expression of Chk1 in Caco-2 cells, 50 nM siRNA for Chk1 (Cell Signaling, Beverly, MA, USA) was transfected into Caco-2 cells by using Lipofectamine RNAiMAX Reagent (Invitrogen). After culturing for 2 days, the Caco-2 cells were transferred to Transwell chambers (diameter, 6.5 mm; pore size, 0.4 μm; Corning) for TER assay.

### Immunofluorescence analysis

Caco-2 cells were seeded in Transwell chambers (diameter, 12 mm; pore size, 0.4 μm; Corning) at a density of 1.5 × 10^5^ cells/well and cultured for 10 to 14 days. After treatment with 1 μM daunorubicin and rebeccamycin for 48 h, Caco-2 cells were fixed with 4% paraformaldehyde for 15 min, and permeabilized with 0.1% Triton X-100 in PBS for 5 min. Cells were then blocked with 1% BSA in TBS buffer (20 mM Tris—HCl [pH 7.4], 40 mM NaCl) containing 0.05% Tween-20 (T-TBS) for 1 h, followed by incubation with anti-p-Chk1 antibody in 1% BSA in T-TBS for 1 h. After incubation with secondary fluorescence antibodies and DAPI (Sigma-Aldrich) for 1 h, immunofluorescence images were captured under a fluorescence microscope (Keyence, Tokyo, Japan).

### Statistical analysis

Data are presented as mean ± SD. Dunnett's or Tukey’s test was used for statistical analyses. A *P* value of less than 0.05 was considered indicative of statistical significance.

## Results

### Daunorubicin and rebeccamycin enhance TJ barrier function in Caco-2 cell monolayer

**Fig 1:**
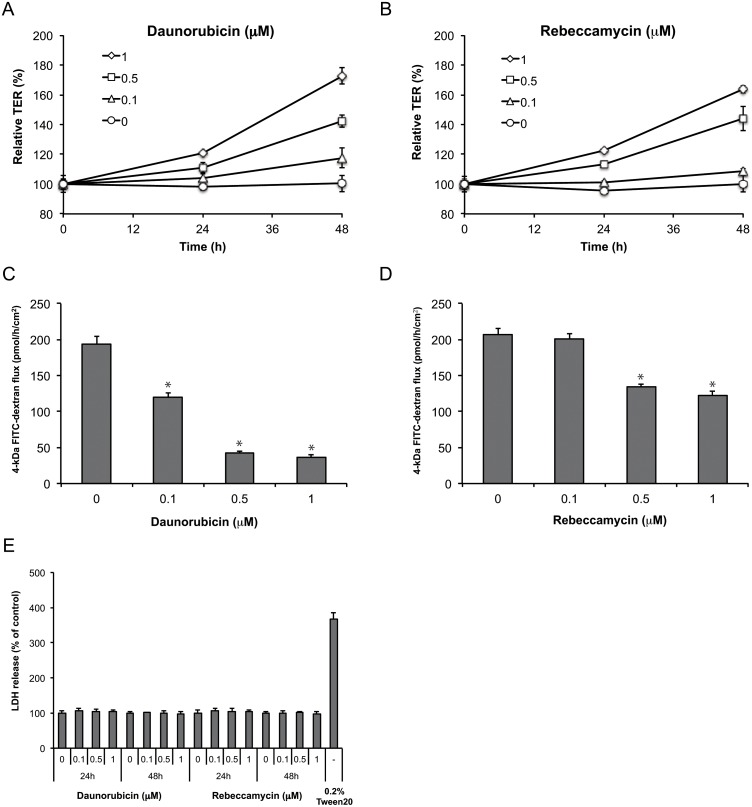
Daunorubicin and rebeccamycin enhance tight junction barrier function in Caco-2 cell monolayer. (A-D) Caco-2 cells were treated with vehicle or 0.1, 0.5, or 1 μM daunorubicin (A, B) or rebeccamycin (C, D) on the apical and basolateral sides. Transepithelial electrical resistance (TER) value was measured every 24 h (A, C), and the flux of 4-kDa FITC—dextran was measured at 48 h (B, D). TER is presented as a percentage of the TER value at 0 h. (E) Caco-2 cells were treated with vehicle or 0.1, 0.5, or 1 μM daunorubicin or rebeccamycin for 24 h and 48 h, and then lactate dehydrogenase (LDH) release was measured; 0.2% Tween 20 served as the positive control. LDH release is presented as a percentage of the LDH released by the vehicle-treated cells. Data are presented as mean ± SD (n = 3). *P < 0.05 vs. vehicle-treated group, as determined by Dunnett's test.

To investigate the effects of daunorubicin and rebeccamycin on epithelial barrier function, we used the human intestinal Caco-2 cell line, which is a widely employed model for functional and molecular analysis of intestinal epithelia. We first examined the effects of daunorubicin and rebeccamycin on epithelial barrier function by using TER analysis. Caco-2 cells were first cultured in Transwell chambers for 10 to 14 days. Daunorubicin or rebeccamycin was then added, and the cells were cultured for another 24 or 48 h before TER analysis. Daunorubicin or rebeccamycin induced an increase in TER compared with control in a dose- and time-dependent manner (Fig 1A and 1B). Daunorubicin and rebeccamycin also increased TER values in the human intestinal T84 cell line (Figure A and B in S1 File). In addition, the anti-tumor drugs cisplatin and mitomycin C induced an increase in TER in Caco-2 cells (S1 Fig).

We next examined the effect of daunorubicin and rebeccamycin on paracellular flux. After treatment with daunorubicin or rebeccamycin for 48 h, we added 4-kDa FITC—dextran, as a paracellular tracer, to the apical side of the Caco-2 cell layer. After incubation for 1 h, we measured the amount of 4-kDa FITC—dextran that had passed through to the basolateral side of the cell layer. Daunorubicin and rebeccamycin decreased paracellular flux of 4-kDa FITC—dextran in a dose-dependent manner (Fig 1C and 1D). Combined with the results of TER analysis, these data suggest that daunorubicin and rebeccamycin enhance the TJ barrier function in Caco-2 cell monolayer. Daunorubicin and rebeccamycin were not cytotoxic at the concentrations at which they enhanced TJ barrier function (Fig 1E).

### Daunorubicin and rebeccamycin significantly ameliorate inflammatory cytokine-mediated attenuation of TJ barrier function

**Fig 2:**
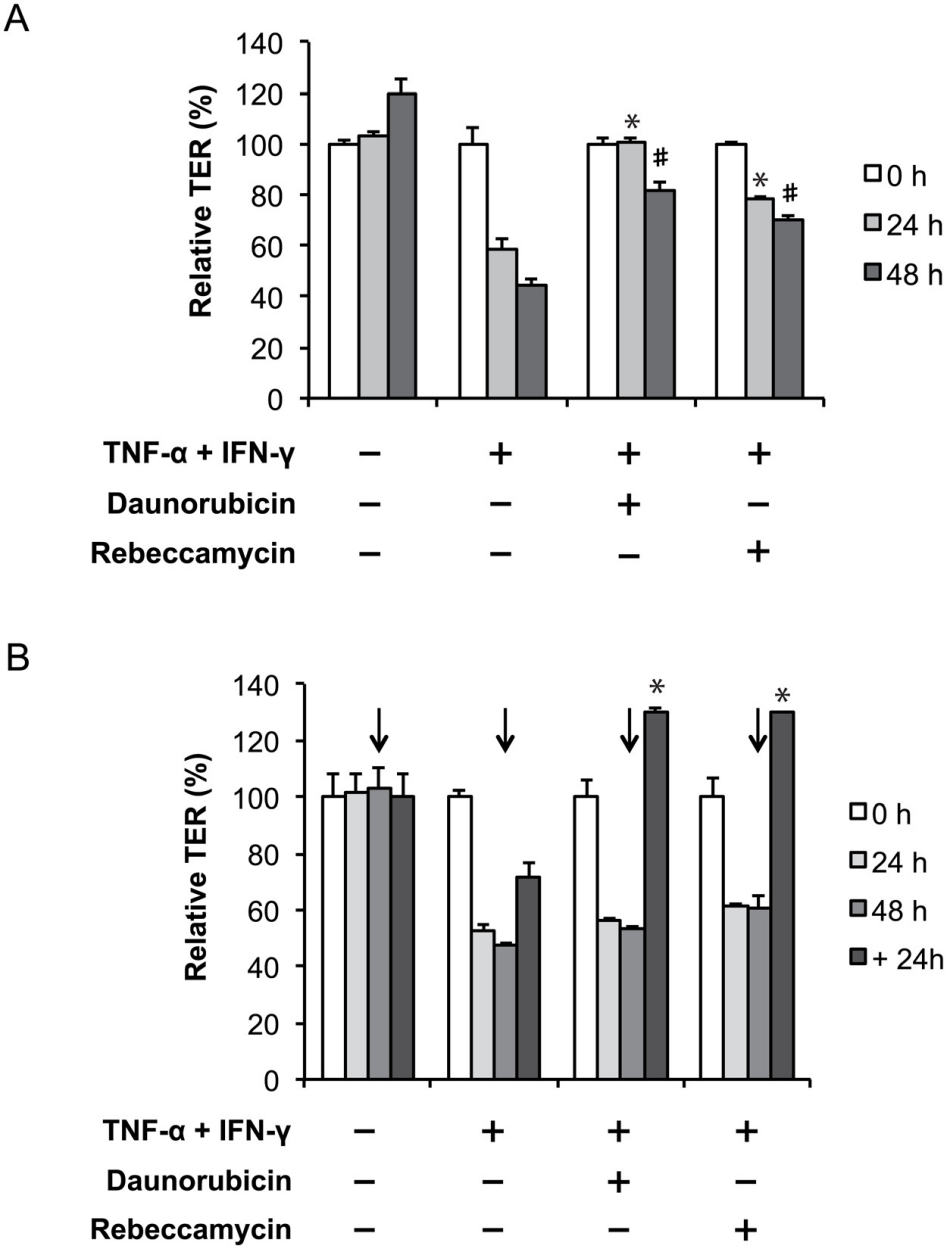
Daunorubicin and rebeccamycin restore the tight junction barrier attenuation induced by TNF-α and IFN-γ. (A) Caco-2 cells were incubated with vehicle or 10 ng/ml TNF-α and IFN-γ in the absence or presence of 1 μM daunorubicin or rebeccamycin. After incubation, TER value was measured every 24 h. (B) Caco-2 cells were incubated with vehicle or 10 ng/ml TNF-α and IFN-γ for 48 h. After incubation, the medium was replaced with fresh medium in the containing or lacking of 1 μM daunorubicin or rebeccamycin, and the cells were cultured for an additional 24 h. TER value was measured every 24 h. Arrows indicate the point of removal of TNF-α and IFN-γ. Data are presented as mean ± SD (n = 3). *P* < 0.05 vs. TNF-α and IFN-γ treatment for 24 h (*) or 48 h (#), as determined by Tukey’s test.

Inflammatory cytokines attenuate intestinal TJ integrity, resulting in an increase in paracellular permeability, which triggers chronic inflammation in the intestine [14, 20]. To investigate the effect of daunorubicin or rebeccamycin on inflammatory cytokine-mediated attenuation of TJ integrity, we treated Caco-2 cells with TNF-α or IFN-γ, which are highly expressed in the chronically inflamed intestine. Daunorubicin and rebeccamycin significantly suppressed TNF-α and IFN-γ-mediated attenuation of TJ integrity, as assessed by TER (Fig 2A). Furthermore, treatment with daunorubicin or rebeccamycin restored inflammatory cytokine-mediated TJ barrier function attenuation earlier than did treatment with vehicle (Fig 2B). These results indicate that the enhancement of TJ barrier function by daunorubicin and rebeccamycin ameliorates the dysfunction of TJ integrity induced by inflammatory cytokines.

### Daunorubicin and rebeccamycin increase claudin-5 expression

**Fig 3:**
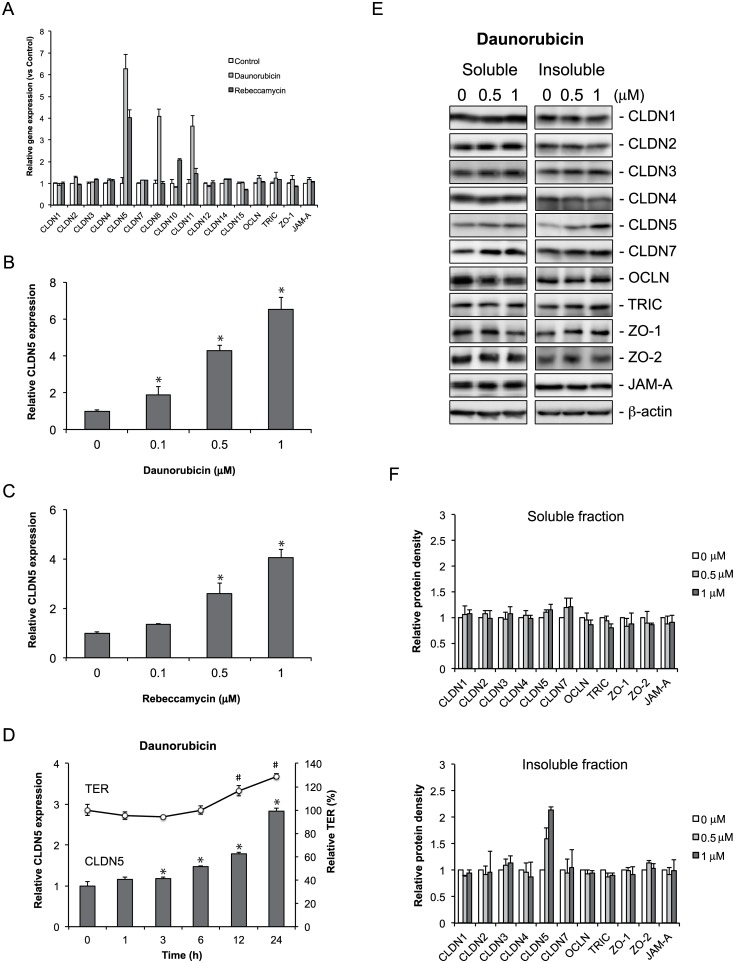
Daunorubicin and rebeccamycin increase claudin-5 expression in Caco-2 cells. (A) Caco-2 cells were treated with vehicle or 1 μM daunorubicin or rebeccamycin for 48 h. Cell lysates were collected and subjected to qRT-PCR analysis for claudin (CLDN), occludin (OCLN), tricellulin (TRIC), zonula occludens (ZO)-1, and junctional adhesion molecule (JAM)-A. (B, C) Cells were treated with vehicle or 0.1, 0.5, or 1 μM daunorubicin (B) or rebeccamycin (C) for 48 h, and cell lysates were collected and subjected to qRT-PCR analysis for claudin-5 expression. Claudin-5 expression is presented relative to that obtained with vehicle treatment. *P < 0.05 vs. vehicle-treated group, as determined by Dunnett's test. (D) Cells were treated with 1 μM daunorubicin, and cell lysates were collected at 0, 1, 3, 6, 12, and 24 h and subjected to qRT-PCR analysis for claudin-5 expression. Time course changes in transepithelial electrical resistance (TER) after treatment with 1 μM daunorubicin are also shown. *P* < 0.05 relative to TER (#) or CLDN5 expression (*) at 0 h, as determined by Dunnett's test. (E) Cells were treated with vehicle or 0.5 or 1 μM daunorubicin for 48 h. Detergent (Triton-X)-soluble and -insoluble fractions were collected and immunoblotted for claudin-1, -2, -3, -4, -5, -7, occludin, and tricellulin, ZO-1, ZO-2, and JAM-A. β-actin served as the loading control. Relative protein density was calculated as the ratio of the protein density to the density of vehicle (F). Data are presented as mean ± SD (n = 3).

Because epithelial TJ barrier function is controlled by the various TJ components, we examined the effect of daunorubicin and rebeccamycin on the transcription of TJ components. qRT-PCR analysis showed that daunorubicin mainly increased the expression of claudin-5, -8, and -11, whereas rebeccamycin significantly increased the expression of claudin-5 and -10 (Fig 3A). Thus, claudin-5 expression was increased by both daunorubicin and rebeccamycin treatment. This increase in claudin-5 expression was dose dependent for both daunorubicin and rebeccamycin (Fig 3B and 3C). Claudin-5 expression was significantly increased from 3 h after daunorubicin was added to the cells, and TER values were significantly increased from 12 h after daunorubicin was added to the cells (Fig 3D). A similar pattern was observed with rebeccamycin treatment, although the increases in Claudin-5 expression and TER values occurred later than they did with daunorubicin treatment (S2 Fig).

Epithelial TJ barrier function is affected not only by the expression of TJ components, but also by their cellular localization. Therefore, to examine the effect of daunorubicin on the expression and cellular localization of various claudins and other TJ components, we prepared detergent-soluble and -insoluble lysates of cells treated with daunorubicin. Detergent-insoluble lysates contain proteins associated with the actin cytoskeleton, which includes TJ components [21]. The level of claudin-5 protein in the insoluble fraction was elevated with increasing daunorubicin or rebeccamycin concentration (Fig 3E, 3F, Figure A and B in S2 File). Exogenous expression of claudin-5 using a retroviral expression system increased TER in Caco-2 cells (Figure A and B in S3 File). These findings suggest that daunorubicin and rebeccamycin enhanced the TJ barrier function in Caco-2 cell monolayer by elevating claudin-5 expression.

### Chk1 is essential for daunorubicin- and rebeccamycin-induced enhancement of TJ barrier function

**Fig 4:**
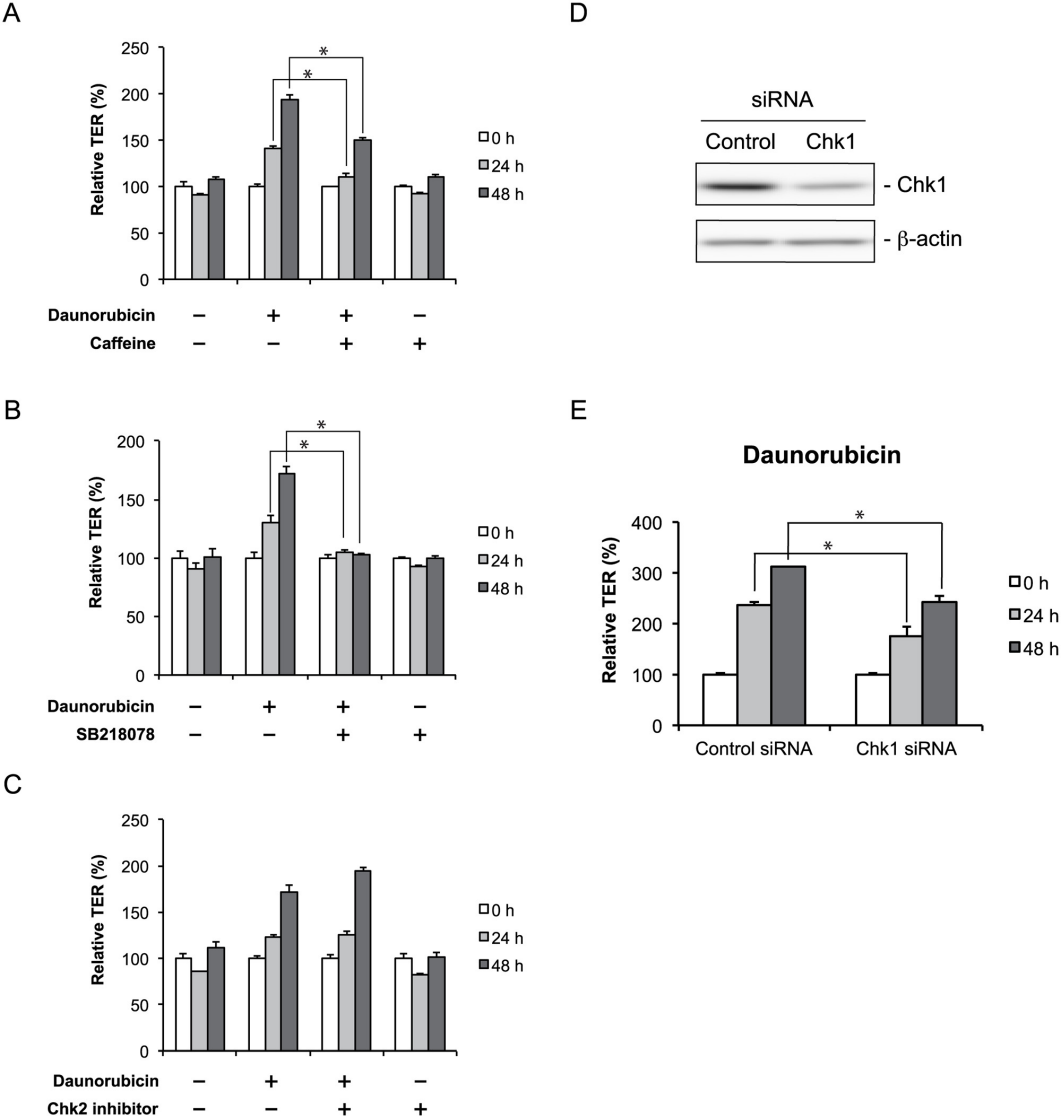
Checkpoint kinase 1 is essential for daunorubicin-mediated enhancement of tight junction barrier function. (A) Caco-2 cells were treated with vehicle or 1 μM daunorubicin for 48 h in the absence or presence of 5 mM caffeine. After incubation, TER value was measured every 24 h. (B, C) Cells were treated with vehicle or 1 μM daunorubicin for 48 h in the absence or presence of SB218078 (B) or Chk2 inhibitor (C). After incubation, TER value was measured every 24 h. (D) Cells were transfected with 50 nM control or Chk1 siRNA and incubated for 7 days. Chk1 protein was detected by immunoblotting. β-actin was used as the loading control. (E) Cells were transfected with 50 nM control siRNA or Chk1 siRNA, incubated for 7 days, and then treated with 1 μM daunorubicin for 48 h. TER value was measured every 24 h. Data are presented as mean ± SD (n = 3). *P < 0.05, as determined by Tukey’s test.

Daunorubicin and rebeccamycin mainly inhibit DNA replication by damaging the DNA in tumors [17, 18]. We therefore examined the possibility that DNA damage signaling pathways were involved in the enhancement of TJ barrier function induced by daunorubicin and rebeccamycin. To investigate the involvement of ATM and ATR activation in the daunorubicin- and rebeccamycin-mediated effects on TJ barrier function, we examined the effects of caffeine, an inhibitor of ATM and ATR, on the activity of daunorubicin and rebeccamycin. Caffeine significantly suppressed the increase in TER induced by daunorubicin or rebeccamycin treatment (Fig 4A and Figure A in S4 File).

We next used Chk1 and Chk2 inhibitors to determine whether Chk1 and Chk2 play a role in daunorubicin- or rebeccamycin-mediated enhancement of TJ barrier function. The Chk1 inhibitor SB218078 markedly suppressed the increase in TER induced by daunorubicin or rebeccamycin (Fig 4B and Figure B in S4 File); however, a Chk2 inhibitor did not (Fig 4C and Figure C in S4 File). These results suggest that daunorubicin and rebeccamycin both increase TJ barrier function via Chk1 activation. To confirm the contribution of Chk1, we examined the effects of Chk1 siRNA on daunorubicin- or rebeccamycin-mediated enhancement of TJ barrier function. Immunoblot analysis showed that Chk1 protein expression was decreased by Chk1 siRNA compared with control (Fig 4D). The increase in TER induced by daunorubicin and rebeccamycin was significantly suppressed by treatment with Chk1 siRNA compared with control siRNA (Fig 4E and Figure D in S4 File). These data indicate that Chk1 plays a crucial role in daunorubicin- and rebeccamycin-mediated enhancement of TJ barrier function.

### Daunorubicin and rebeccamycin activate DNA damage signaling in the nucleus

**Fig 5:**
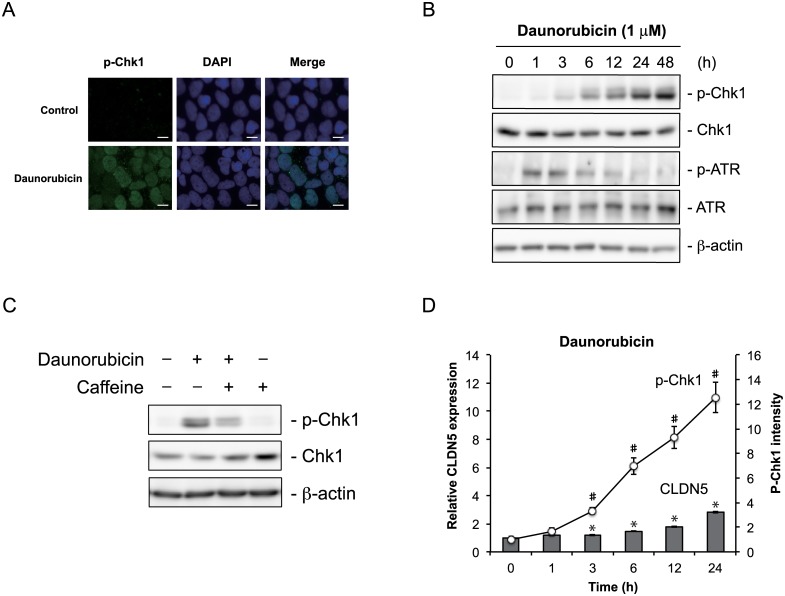
Daunorubicin stimulates DNA damage signaling in Caco-2 cells. (A) Caco-2 cells were treated with or without 1 μ M daunorubicin for 48 h, and then fixed and stained with anti-p-Chk1 antibody and DAPI. Images were collected under a fluorescence microscope. Scale bar = 10 μm. (B) After cells were treated with or without 1 μ M daunorubicin, whole cell extracts were collected at 1, 3, 6, 12, 24, and 48 h and immunoblotted for Chk1, p-Chk1, ATR, and p-ATR. β-actin served as the loading control. (C) Cells were treated with vehicle or 1 μM daunorubicin for 48 h in the absence or presence of 5 mM caffeine. After incubation, cell lysates were collected and subjected to immunoblot analysis for Chk1 and p-Chk1. β-actin served as the loading control. (D) Cells were treated with 1 μM daunorubicin and cell lysates were collected at 0, 1, 3, 6, 12, and 24 h. The cell lysates were then subjected to immunoblot analysis for p-Chk1. Intensity of p-ChK1 was quantitated by means of densitometric analysis. Time course changes in claudin-5 expression after treatment with 1 μM daunorubicin are also shown. Data are presented as mean ± SD (n = 3). *P* < 0.05 relative to p-Chk1 (#) or claudin-5 expression (*) at 0 h, as determined by Dunnett's test.

Next, we determined the effect of daunorubicin and rebeccamycin on the cellular localization of activated Chk1 by means of immunofluorescence analysis. Activated Chk1 in daunorubicin- or rebeccamycin-treated cells was co-localized with DAPI-stained areas, indicating the presence of activated Chk1 in the nucleus of these cells (Fig 5A and Figure A in S5 File). We next investigated the time course of Chk1 activation following daunorubicin treatment. Activation of Chk1 was detectable at 3 h following daunorubicin treatment and this activation gradually increased over time. Preceding Chk1 activation, ATR was activated and this activity decreased over time (Fig 5B). Also, Chk1 activation was dependent on ATR activity (Fig 5C). These results indicate that daunorubicin stimulates the ATR—Chk1 pathway. Chk1 activation started to increase 3 h after daunorubicin was added to the cells and claudin-5 expression was also significantly increased from 3 h after daunorubicin treatment (Fig 5D). A similar pattern was observed with rebeccamycin treatment, although the increases in Chk1 activation and claudin-5 expression occurred later than they did with daunorubicin treatment (Figure B–D in S5 File). Together, these results imply that claudin-5 expression is regulated by Chk1 activation.

### Enhancement of claudin-5 expression by daunorubicin and rebeccamycin is controlled by Chk1 activation

**Fig 6:**
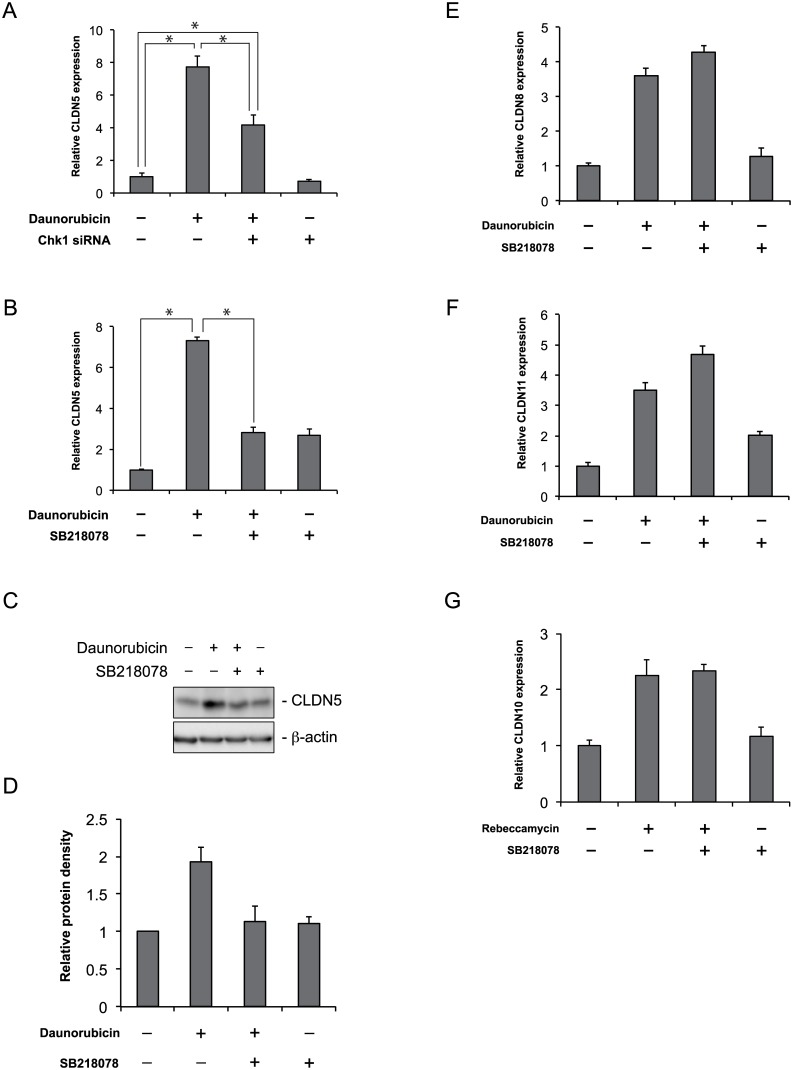
Checkpoint kinase 1 activation is important for daunorubicin-mediated enhancement of claudin-5 expression. Caco-2 cells were treated with vehicle, 1 μM daunorubicin, or rebeccamycin for 48 h in the absence or presence of Chk1 siRNA (A) or 2 μM SB218078 (B, E, F, and G). After incubation, claudin-5 (A, B), claudin-8 (E), claudin-11 (F), or claudin-10 (G) expression was measured with qRT-PCR. Data are presented as mean ± SD (n = 3). *P < 0.05, as determined by Tukey’s test. (C) Cells were treated with vehicle or 1 μM daunorubicin for 48 h in the absence or presence of 2 μM SB218078. After incubation, cell lysates were collected and subjected to immunoblot analysis for claudin-5. β-actin served as the loading control. Relative protein density was calculated as the ratio of the protein density to the density of vehicle (D). Data are presented as mean ± SD (n = 3).

To confirm the link between Chk1 activation and claudin-5 expression, we investigated the effect of Chk1 siRNA on the daunorubicin- or rebeccamycin-induced increase in claudin-5 expression. Chk1 siRNA significantly inhibited the daunorubicin- or rebeccamycin-induced increase in claudin-5 expression (Fig 6A and Figure A in S6 File). Furthermore, the Chk1 inhibitor SB218078 suppressed the daunorubicin- or rebeccamycin-induced increase in claudin-5 mRNA and protein expression (Fig 6B–6D; Figure B–D in S6 File). These results indicate that the elevation of claudin-5 expression induced by daunorubicin and rebeccamycin is dependent on Chk1 activation. Daunorubicin or rebeccamycin increased not only the expression of claudin-5, but also the expression of claudin-8 and claudin-11, or claudin-10, respectively (Fig 3A). We therefore examined whether Chk1 activation was involved in the daunorubicin or rebeccamycin-mediated enhancement of claudin-8 and claudin-11, or claudin-10 expression. In contrast to its inhibitory effect on the daunorubicin or rebeccamycin-mediated increase in claudin-5 expression, the Chk1 inhibitor did not affect the enhancement of claudin-8 and claudin-11, or claudin-10 expression (Fig 6E–6G).

## Discussion

**Fig 7:**
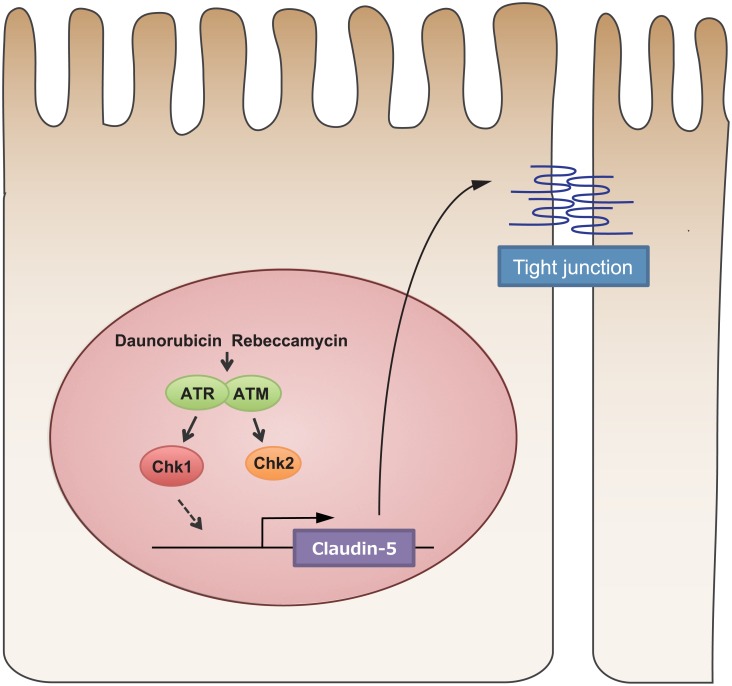
Schematic overview of DNA damage-mediated enhancement of tight junction barrier function in Caco-2 cells.

In the present study, we evaluated the effects of daunorubicin and rebeccamycin on intestinal epithelial TJ integrity and revealed not only that daunorubicin and rebeccamycin enhanced TJ barrier function in Caco-2 cell monolayer, but also that they ameliorated inflammatory cytokine-mediated attenuation of TJ integrity. Therefore, we hypothesize that daunorubicin and rebeccamycin activate ATR in the nucleus, which results in activation of the downstream molecule Chk1. Activated Chk1 then increases the expression of claudin-5, which then translocates to the TJ where it enhances TJ barrier function (Fig 7). Of note, daunorubicin and rebeccamycin were not cytotoxic at the concentrations at which they enhanced TJ barrier function, and they enhanced TJ barrier function in a dose-dependent manner. Thus, the effect of daunorubicin or rebeccamycin on TJ-barrier function is not a result of their cytotoxicity. Corresponding observations with other DNA damage inducers (i.e., cisplatin and mitomycin C) and another intestinal cell line (i.e., T84) lend support to the hypothesis that certain DNA damage inducers enhance TJ barrier function in intestinal cells. Several studies have demonstrated that stressors such as oxidants, alcohol, and heavy metals influence epithelial barrier function, leading to increased intestinal permeability, but these stressors disrupt epithelial barrier function by eliciting reduction or replacement of TJ components [22–24]. In contrast, our observations indicate that DNA damage, while not enough to exert cytotoxicity, can enhance TJ barrier function in intestinal cells. Therefore, epithelial cells that have sustained irreversible DNA damage or that are under severe environmental stress have a compromised barrier function and may go into apoptosis, whereas those under moderate external stress or environmental changes may modulate their TJ integrity to maintain internal homeostasis [25].

Chk1 is a critical mediator of signal transduction in the DNA damage response where it triggers cell cycle checkpoints and contributes to preserving genome integrity. The ATM—Chk2 pathway principally responds to DNA double-strand breaks, whereas the ATR—Chk1 pathway responds to a broad range of DNA abnormalities caused by ultra-violet light, DNA replication block, virus infection, interstrand DNA crosslinking, and double-strand break end resection [26–30]. Although the major function of Chk1 is to trigger the cell cycle checkpoint response after DNA damage, it also controls a number of other cellular functions, including DNA damage repair, gene transcription, and embryo development [31–33]. The functions of Chk1 in the cell cycle checkpoints overlap with those of Chk2 (e.g., Cdc25 and p53 regulation). However, Chk1 and Chk2 have specific substrates and thereby exert different cellular functions [3]. In the present study, Chk1, but not Chk2, activation in response to DNA damage enhanced TJ barrier function in intestinal cells, and Chk1 activation increased the expression of the TJ-sealing component claudin-5. These findings suggest that Chk1 controls the TJ barrier function by mediating the expression of specific claudins; however, additional studies are needed to elucidate the underlying mechanisms of Chk1-mediated claudin-5 expression.

A number of studies suggest that claudin-5 is a major determinant of the properties of the endothelial barrier, especially in the blood—brain barrier and blood—retina barrier. Claudin-5-knockout mice exhibit a leaky brain microvascular endothelial cell phenotype that leads to death within 1 day of birth [34]. Administration of claudin-5 siRNA results in size-selective, transient, reversible modulation of the blood—retina barrier in mouse [35]. Claudin-5 is expressed in vascular endothelia, as well as in uterine epithelium, stomach surface and glands, and throughout the intestine, without gradation along the crypt-to-villus surface axis [36, 37]. Amasheh et al. have shown that claudin-5 partly contributes to the paracellular seal in Caco-2 cells [38]. Consistent with these previous studies, exogenous expression of claudin-5 by retroviral transfection distinctly enhanced TJ barrier function in Caco-2 cell monolayer, indicating that claudin-5 contributes to the sealing function of TJs in intestinal cells. Although both daunorubicin and rebeccamycin mainly increased the expression of claudin-5 in Caco-2 cells, daunorubicin also increased the expression of claudin-8 and claudin-11. Previous studies have shown that ectopic claudin-8 expression increases TER and reduces paracellular flux of tracer molecules in MDCK cells [39], and that upregulation of claudin-8 by aldosterone prevents Na^+^ permeability via the paracellular route in distal colon [40]. The sealing function of claudin-11 in knockout mice has also been reported [41, 42]. These results imply that upregulation of claudin-8 and claudin-11 might be associated with enhancement of the TJ barrier function in daunorubicin-treated Caco-2 cells. However, the contributions of these claudins are likely to be negligible because enhancement of TJ barrier function induced by daunorubicin was largely dependent on Chk1 activity, and activated Chk1 regulated claudin-5, not claudin-8, claudin-11, or claudin-10 expression (Fig 6E–6G).

Epithelial TJ barrier function is essential for creating distinct compartments within the body and for controlling paracellular permeability. Therefore, disruption of TJ integrity leads to disruption of tissue homeostasis, which is associated with a number of diseases [6, 10]. For example, a reduced number of TJ sealing strands, frequent strand breaks, and a discontinuous TJ network are observed in patients with intestinal inflammatory disease [43, 44]. These alterations result in disruption of the epithelial barrier, leading to leak flux diarrhea and facilitate uptake of noxious agents, which further promote intestinal inflammation and mucosal injury. This continual immune activation provokes systemic immune dysregulation, causing persistent DNA damage [45]. The expression and subcellular distribution of claudins differs in patients with intestinal inflammatory disease [14]; claudin-2, -5, -8 and claudin-2, -3, -5 expression is altered in the intestine of patients with Crohn's disease and celiac disease, respectively [44, 46], suggesting that claudin dysfunction may be an important symptom of these diseases. Therapeutically strengthening epithelial barrier function may be a promising way to improve the symptoms of intestinal inflammatory diseases [14, 43], although anti-inflammatory strategies remain the predominant treatment option for these diseases [47]. Further detailed studies of the function and regulation of TJ components, including those of claudin-5, will provide opportunities for the development of effective approaches for treating inflammatory diseases.

In the present study, we demonstrated that daunorubicin- or rebeccamycin-induced DNA damage enhanced intestinal TJ barrier function via Chk1 activation. The physiological significance of DNA damage-induced enhancement of TJ barrier function remains unclear, but our observations suggest that TJ barrier function is less a “static” function than it is a “dynamic” function that responds to external nocuous stimulation. Further analysis of the TJ barrier function will provide new clues for understanding the link between external stress and the response of the TJ barrier function.

## Supporting Information

S1 Fig Cisplatin and mitomycin C enhance TER in Caco-2 cell monolayer. Caco-2 Cells were treated with vehicle or 1 μM cisplatin or mitomycin C. TER value was measured at 24 and 48 h. TER is presented as a percentage of the TER value at 0 h. Data are presented as mean ± SD (n = 3). *P < 0.05 vs. 0 h, as determined by Dunnett's test.
(EPS)

S2 Fig Time course changes in claudin-5 expression after rebeccamycin treatment. Cells were treated with 1 μM rebeccamycin, and cell lysates were collected at 0, 1, 3, 6, 12, and 24 h and subjected to qRT-PCR analysis. Data are presented as mean ± SD (n = 3). Time course changes in TER value after treatment with 1 μM rebeccamycin are also shown. *P* < 0.05 relative to TER (#) or CLDN5 expression (*) at 0 h, as determined by Dunnett's test.
(EPS)

S1 File Daunorubicin and rebeccamycin enhance tight junction barrier function in T84 cells. T84 cells were treated with vehicle or 0.1, 0.5, or 1 μM daunorubicin (Figure A) or rebeccamycin (Figure B) on the apical and basolateral side. TER value was measured at 24 h. TER is presented as a percentage of the TER value at 0 h. Data are presented as mean ± SD (n = 3). *P < 0.05 vs. vehicle-treated group, as determined by Dunnett's test.
(EPS)

S2 File Rebeccamycin increases claudin-5 protein expression in Caco-2 cells. (Figure A) Cells were treated with vehicle or 0.5 or 1 μM rebeccamycin for 48 h. Detergent-soluble and -insoluble fractions were collected and immunoblotted for claudin-1, -2, -3, -4, -5, -7, occludin, and tricellulin. β-actin served as the loading control. Relative protein density was calculated as the ratio of the protein density to the density of vehicle (Figure B). Data are presented as mean ± SD (n = 3).
(EPS)

S3 File Exogenous claudin-5 expression increases TER in Caco-2 cells. (Figure A) Expression of claudin-1, -3, -4, and -5 in Caco-2 cells exogenously expressing claudin-5 (Caco-2/CLDN5) was assessed by immunoblot analysis. β-actin was used as the loading control. (Figure B) Caco-2/vector and Caco-2/CLDN5 cells were cultured in Transwell chambers for 10 days and then TER value was measured. TER is presented as a percentage of the TER value for Caco-2/vector. Data are presented as mean ± SD (n = 3).
(EPS)

S4 File Chk1 activation is important for rebeccamycin-mediated enhancement of claudin-5 expression. (Figure A) Caco-2 cells were treated with vehicle or 1 μM rebeccamycin for 48 h in the absence or presence of 5 mM caffeine. After incubation, TER value was measured every 24 h. (Figure B, C) Cells were treated with vehicle or 1 μM rebeccamycin for 48 h in the absence or presence of SB218078 (Figure B) or Chk2 inhibitor (Figure C). After incubation, TER value was measured every 24 h. (Figure D) Cells were transfected with 50 nM control siRNA or Chk1 siRNA, incubated for 7 days, and then treated with 1 μM rebeccamycin for 48 h. TER was measured every 24 h. Data are presented as mean ± SD (n = 3). *P < 0.05, as determined by Tukey’s test.
(EPS)

S5 File Rebeccamycin stimulates DNA damage signaling in Caco-2 cells. (Figure A) Caco-2 cells were treated with or without 1 μ M rebeccamycin for 48 h, and then fixed and stained with anti-p-Chk1 antibody and DAPI. Images were viewed under a confocal microscope. Scale bar = 10 μm (Figure B) Cells were treated with or without 1 μ M rebeccamycin and whole cell extracts were collected at 1, 3, 6, 12, 24, and 48 h and immunoblotted for Chk1, p-Chk1, ATR, and p-ATR. β-actin served as the loading control. (Figure C) Cells were treated with vehicle or 1 μM rebeccamycin for 48 h in the absence or presence of 5 mM caffeine. After incubation, cell lysates were collected and subjected to immunoblot analysis for Chk1 and p-Chk1. β-actin served as the loading control. (Figure D) Cells were treated with 1 μM rebeccamycin, and cell lysates were collected at 0, 1, 3, 6, 12, and 24 h. Cell lysates were then subjected to immunoblot analysis for p-Chk1. Intensity of p-ChK1 was quantitated by densitometric analysis. Data are presented as mean ± SD (n = 3). Time course changes in claudin-5 expression after treatment with 1 μM rebeccamycin are also shown. *P* < 0.05 relative to p-Chk1 (#) or CLDN5 expression (*) at 0 h, as determined by Dunnett's test.
(EPS)

S6 File Chk1 activation is important for rebeccamycin-mediated enhancement of claudin-5 expression. Caco-2 cells were treated with vehicle or 1 μM rebeccamycin for 48 h in the absence or presence of Chk1 siRNA (Figure A) or SB218078 (Figure B). After incubation, claudin-5 expression was measured with qRT-PCR. Data are presented as mean ± SD (n = 3). *P < 0.05, as determined by Tukey’s test. (Figure C) Cells were treated with vehicle or 1 μM rebeccamycin for 48 h in the absence or presence of 2 μM SB218078. After incubation, cell lysates were collected and subjected to immunoblot analysis for claudin-5. β-actin served as the loading control. Relative protein density was calculated as the ratio of the protein density to the density of vehicle (Figure D). Data are presented as mean ± SD (n = 3).
(EPS)

S1 Table Sequences of primers used in quantitative reverse transcription polymerase chain reaction analysis. (EPS)
